# BE-FLARE: a fluorescent reporter of base editing activity reveals editing characteristics of APOBEC3A and APOBEC3B

**DOI:** 10.1186/s12915-018-0617-1

**Published:** 2018-12-28

**Authors:** Matthew A. Coelho, Songyuan Li, Luna Simona Pane, Mike Firth, Giovanni Ciotta, Jonathan D. Wrigley, Maria Emanuela Cuomo, Marcello Maresca, Benjamin J. M. Taylor

**Affiliations:** 1 Discovery Sciences, IMED Biotech Unit, AstraZeneca, Cambridge, UK; 2 Discovery Sciences, IMED Biotech Unit, AstraZeneca, Gothenburg, Sweden

**Keywords:** Base editing, Fluorescent reporter, CRISPR/Cas9, APOBEC, Gene editing

## Abstract

**Background:**

Base Editing is a precise genome editing method that uses a deaminase-Cas9 fusion protein to mutate cytidine to thymidine in target DNA in situ without the generation of a double-strand break. However, the efficient enrichment of genetically modified cells using this technique is limited by the ability to detect such events.

**Results:**

We have developed a Base Editing FLuorescent Activity REporter (BE-FLARE), which allows for the enrichment of cells that have undergone editing of target loci based on a fluorescence shift from BFP to GFP. We used BE-FLARE to evaluate the editing efficiency of APOBEC3A and APOBEC3B family members as alternatives deaminase domains to the rat APOBEC1 domain used in base editor 3 (BE3). We identified human APOBEC3A and APOBEC3B as highly efficient cytidine deaminases for base editing applications with unique properties.

**Conclusions:**

Using BE-FLARE to report on the efficiency and precision of editing events, we outline workflows for the accelerated generation of genetically engineered cell models and the discovery of alternative base editors.

**Electronic supplementary material:**

The online version of this article (10.1186/s12915-018-0617-1) contains supplementary material, which is available to authorized users.

## Background

Experimental and therapeutic modification of genomic DNA has become a more rapid and efficient process due to the development of CRISPR-Cas-based technologies. Base editing is a recently developed derivative of CRISPR-Cas-mediated genome editing [1, 2]. The third iteration of the Base Editor protein (BE3) is a fusion of three enzymes: rat APOBEC1 cytidine deaminase, Cas9 D10A nickase, and uracil DNA glycosylase inhibitor (UGI) [1]. This multi-enzyme complex can introduce high-frequency C to T mutations (or G to A on the complementary strand) through enzymatic deamination of cytidine to uracil at the targeted locus. Replication across the uracil will lead to incorporation of a thymidine at this position due to the misrecognition of uracil as thymidine by DNA polymerases. The base excision repair pathway enzyme, uracil DNA glycosylase, could recognise and remove the uracil; however, the UGI component in BE3 provides local inhibition of such repair. Cas9 nickase allows for guide RNA-mediated targeting, and through nicking of the non-edited strand, engenders repair using the edited strand as a template [3].

Introduction or correction of mutations using CRISPR-Cas9 generally depends on DNA double-strand breaks and homology-directed repair (HDR) using an exogenous DNA repair template. This can be a very inefficient process, dependent upon the cell type and cell cycle phase [4–6]. Furthermore, DNA double-strand breaks generated by Cas9 are resolved in an unpredictable manner, often leading to undesirable outcomes such as insertions and deletions (InDels) and translocations [7]. Base editing has unique advantages in this respect; independence from DNA double-strand break formation and HDR leads to reduced rates of InDel formation and a high efficiency of editing in a broader range of cellular contexts [3]. However, producing genetically engineered cell models using base editing still depends on single-cell cloning and sequencing of genomic DNA to find successfully edited cells; this is often the rate-limiting step in the procedure, and gains in the efficiency of this process have the potential to greatly reduce timelines in cell model generation.

Fluorescent reporters developed to discriminate between CRISPR-Cas9-mediated HDR or NHEJ events have facilitated the enrichment of cells with desired DNA repair outcomes and led to improvements in increasing HDR rates in genome engineering [8, 9]. In addition, T2A self-cleaving peptide fusions with fluorescent proteins are common for selecting enriched pools of transfected cells in gene editing experiments. However, a system for reporting on base editor point mutation activity in mammalian cells, which allows for edited cell enrichment and refinement of base editor architecture, has yet to be demonstrated. We used base editing to introduce a well-documented single amino acid substitution in enhanced Blue Fluorescent Protein (eBFP) that leads to a spectral shift associated with a transition to Green Fluorescent Protein (GFP) [10]. By fluorescently marking BE-active cells, we quantitatively assessed efficiencies of different BE variants incorporating alternative APOBEC enzymes and demonstrate FACS-based enrichment of genetically modified cells including gene knock-outs and clinically relevant point mutations. We predict that our reporter will expedite cell model generation with base editing.

## Results

### Validation of a Base Editing FLuorescent Activity REporter (BE-FLARE)

**Fig. 1:**
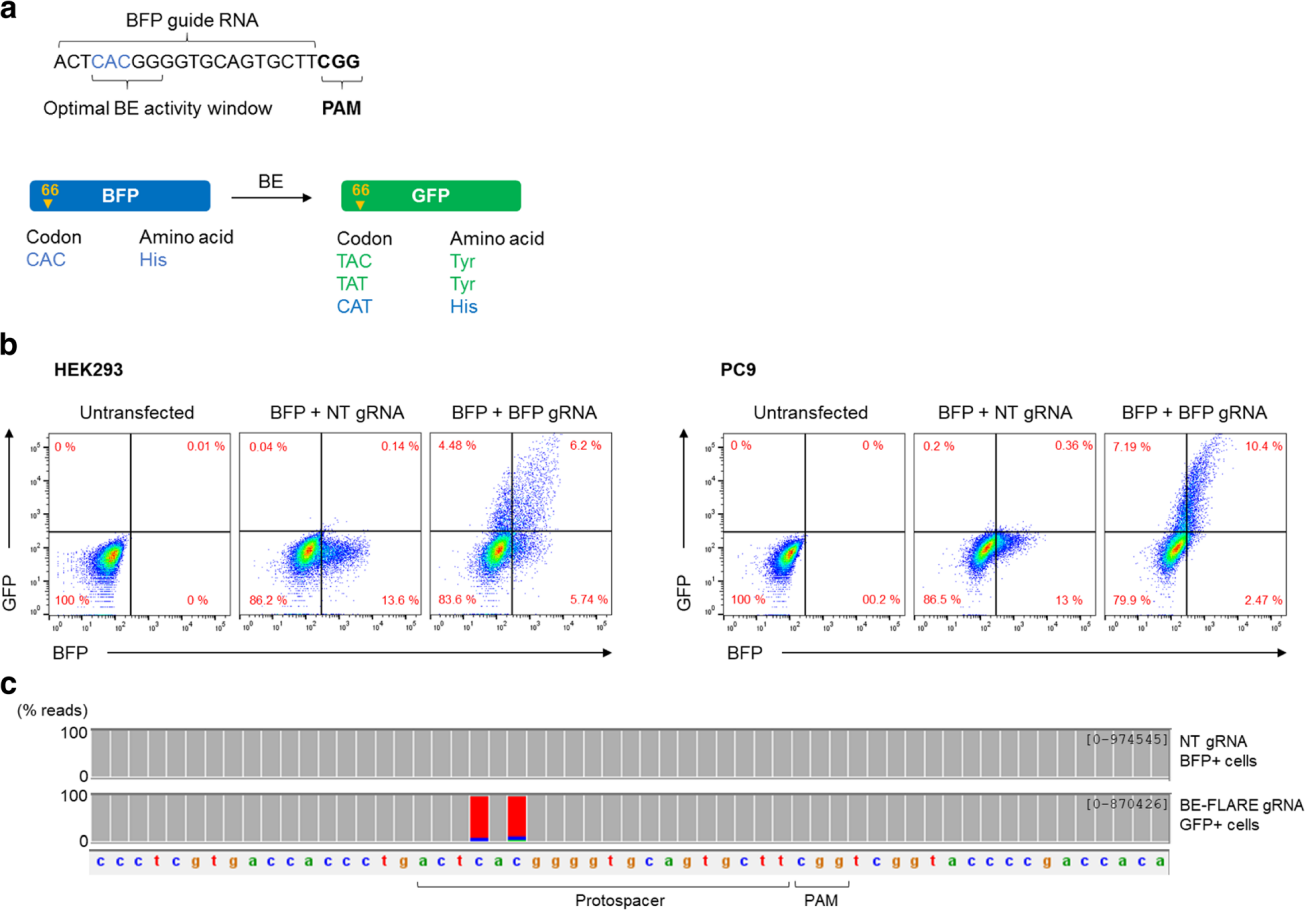
A fluorescent reporter detects base editing activity. **a** Diagram of the BE-FLARE reporter comprised of a modified BFP (BFP) and gRNA sequence used to transition BFP to GFP through base editing (BE). Codon 66 (CAC) encoding histidine is targeted and converted to tyrosine (codons TAT or TAC), resulting in GFP expression. Codon conversion to CAT is synonymous for His, thus the protein remains as BFP. **b** BFP to GFP conversion in HEK293 and PC9 cells. Cells were co-transfected with the BE-FLARE and a plasmid expressing BE3 and either a non-targeting guide (NT-BE) or a BFP targeting guide (BFP-BE). BFP and GFP positive cells were quantified by flow cytometry 72 h after transfection. Data are representative of three independent experiments. **c** PC9 cells from the experiment described in (**b**) were sorted based on BFP (unedited) or GFP fluorescence. Five days later, DNA was extracted for amplicon sequencing of the BFP locus. Data represent a gene browser view of aligned reads in IGV and are representative of two independent experiments. Raw data can be found in Additional file 2

We generated a mammalian expression construct for a version of eBFP that was modified to contain the necessary NGG protospacer adjacent motif (PAM) for *Streptococcus pyrogenes* Cas9, downstream of the target codon histidine 66 (CAC) (Fig. 1a). We termed this reporter the Base Editing FLuorescent Activity REporter (BE-FLARE). We designed a guide RNA (gRNA) targeting eBFP to mutate histidine 66 to tyrosine. Given that BE3 can target neighbouring cytosines in the protospacer within a window of approximately five nucleotides [1], we considered outcomes of other likely editing events. Out of the possible base edits at this codon, two out of three mutations cause a histidine to tyrosine substitution (CAC to TAC or TAT), and the other is synonymous with the wild-type histidine (CAT; Fig. 1a). We introduced a cassette for gRNA expression under the human U6 promoter into the BE3 expression vector to generate a single delivery construct for targeted base editing. Next, we tested BE-FLARE using transient transfections in vitro. We used HEK293 cells and the EGFR-mutant lung cancer cell line, PC9. In both cell lines, GFP signal was detectable after 72 h by flow cytometry only in cells transfected with the construct encoding BE3 together with the gRNA targeting BFP H66, but not a non-targeting control gRNA (non-targeting, NT; Fig. 1b). BE-FLARE was therefore able to report specifically on base editing activity and allows for BE-active cells to be tracked by flow cytometry or microscopy (Additional file 1: Figure S1). To confirm which nucleotides are targeted by BE in the reporter, we performed next-generation amplicon sequencing of BE-FLARE from GFP-positive PC9 cells produced after base editing. As expected, we found that the predominant result of editing codon H66 was CAC->TAT, suggesting that both cytosines within the optimal base editing window are efficiently edited in cells (Fig. 1c).

In addition to transient expression of BE-FLARE, we could stably integrate BE-FLARE using ObLiGaRe-mediated integration into the AAVS1 safe-harbour locus [11], thus allowing for permanent fluorescent demarcation of edited cells. A time-course of digital droplet PCR and microscope imaging of PC9-BE-FLARE cells after editing showed DNA editing of BE-FLARE as early as 18 h and edited cells expressing GFP protein from 48 to 72 h post-transfection (Additional file 1: Figure S2).

### Enrichment of edited cells using BE-FLARE

**Fig. 2:**
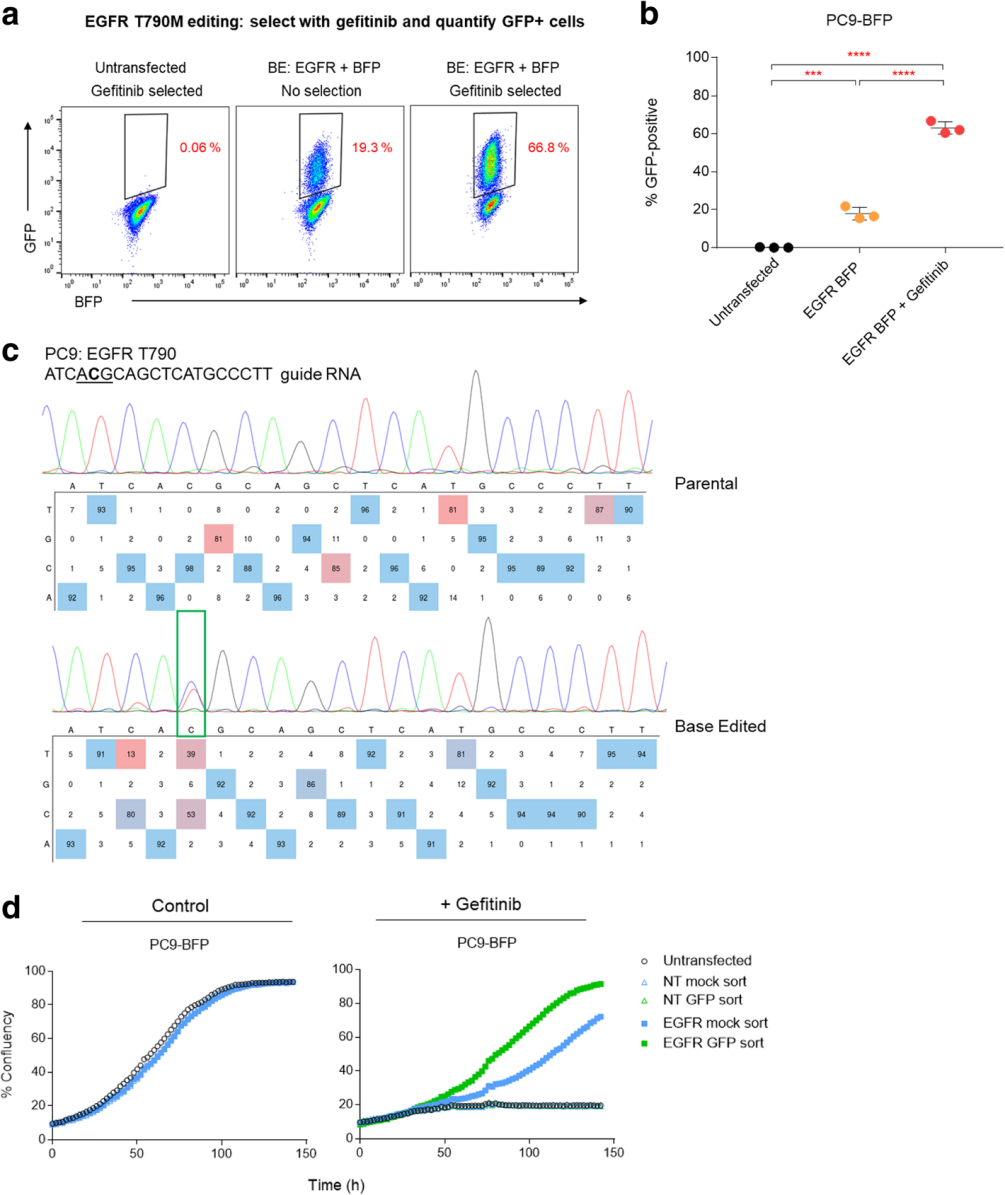
BE-FLARE facilitates reciprocal enrichment of co-edited cells. **a** Enrichment for BFP editing by selection for EGFR T790 M co-editing. PC9 cells stably expressing the BE-FLARE reporter were co-transfected with a construct expressing BE3 and gRNAs targeting EGFR T790 and BFP. Cells were treated with 100 nM gefitinib 72 h after transfection to select for EGFR T790 M mutants. GFP-positive cells were quantified by flow cytometry 4 days after addition of gefitinib. **b** Quantification of three independent experiments from (**a**), with each data point shown and mean represented as a bar. Unpaired Student’s *t* test; ***P < 0.001. **c** Sanger sequencing of the EGFR T790 locus from dual EGFR and BE-FLARE base edited cells after gefitinib selection. Percentage editing was estimated from sequencing chromatograms using EditR [31]. **d** Enrichment for EGFR T790 M editing by selection for BFP co-editing. PC9 cells stably expressing BE-FLARE were co-transfected as above. Cells were mock-sorted (all viable cells) or sorted for GFP expression by FACS 72 h after transfection, and 100 nM gefitinib was added 24 h later. Cell growth was quantified by Incucyte. Data are representative of three independent experiments. Raw data can be found in Additional file 2

We evaluated whether BE-FLARE would allow enrichment for simultaneous co-editing at a secondary locus. As a proof of principle, we generated a cell model with a clinically relevant point mutation; in this instance, the T790 M gate-keeper mutation in human Epidermal Growth Factor Receptor (EGFR), which can be generated by C>T substitution. This mutation confers resistance to the EGFR tyrosine kinase inhibitor gefitinib [12]. Parental PC9 cells are dependent upon oncogenic EGFR signalling and thus are sensitive to gefitinib [13]. We co-transfected PC9-BE-FLARE cells with a BE3 expression construct also encoding a gRNA targeting BFP H66, and a second plasmid expressing a gRNA targeting EGFR T790. Strikingly, selection with gefitinib enriched for GFP-positive cells by ~ 3.5-fold (Fig. 2a and b). We confirmed the successful introduction of the T790 M mutation in the drug-resistant PC9 population by Sanger sequencing (Fig. 2c). In addition to the T790 M base edit, we observed a 5′ bystander C->T mutation within the BE3 activity window. In contrast, the 3′ proximal bystander cytosine remained unedited. Upon inspection of the coding sequence, we noted that the 5′ bystander mutation is synonymous, whereas the 3′ bystander results in a premature stop codon (TAG), which is much less likely to be tolerated in this EGFR-dependent cancer cell line.

In a reciprocal approach, we used flow cytometry to sort for GFP-positive cells following simultaneous base editing of EGFR and BE-FLARE and then quantified gefitinib resistance in GFP-positive versus mock-sorted cells (total viable population; Fig. 2d). We observed a similar co-enrichment using this approach; GFP-positive cells exhibited enhanced levels of resistance to gefitinib relative to mock-sorted controls, with a ~ 4.5-fold increase in cell growth observed after 5 days of gefitinib treatment (Fig. 2d). There was no observable resistance conferred from a non-targeting gRNA in GFP-sorted cells. Thus, an integrated BE-FLARE can be used to enrich for genetic co-editing events at secondary loci.

**Fig. 3:**
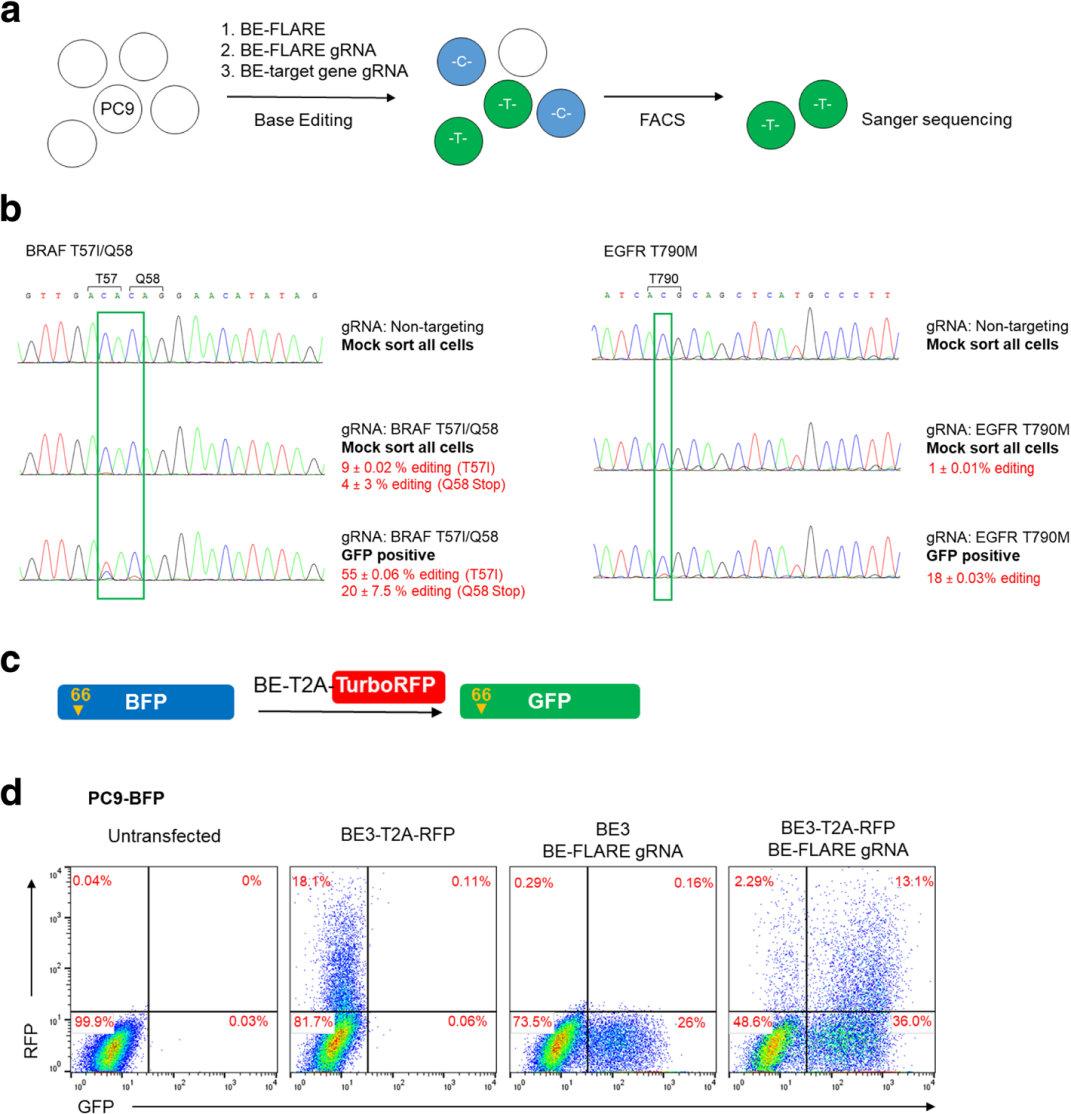
Co-enrichment of editing events using BE-FLARE is superior to selecting transfected cells with BE3-T2A-RFP. **a** Schematic diagram of the enrichment strategy for genomic co-editing events using the BE-FLARE reporter. **b** Co-selection with transient expression of BE-FLARE. PC9 cells were co-transfected with BE-FLARE and a plasmid expressing BE3 and gRNAs targeting BFP and either BRAF T57/Q58 (left) or EGFR T790 (right). Cells were mock-sorted (all viable cells) or sorted for GFP expression by FACS 72 h after transfection and grown for 120 h before harvesting genomic DNA for Sanger sequencing. Cells transfected with non-targeting gRNA served as a negative control reference. Percentage editing was estimated from sequencing chromatograms using EditR [31]. Data are expressed as the mean ± SD of two independent experiments. **c** Schematic of the methodology used to monitor base editing efficiency and BE3 expression using a BE3-T2A-TurboRFP construct (BE3-RFP). **d** BE-FLARE is superior to BE3-T2A-TurboRFP in marking BE3-active cells. PC9 cells stably expressing BE-FLARE were transfected with BE3 or BE3-T2A-turboRFP. Cells were analysed by flow cytometry 72 h after transfection to quantify GFP (edited) and RFP-expressing cells. Data are representative of two independent experiments. Raw data can be found in Additional file 2

We next determined whether we could use BE-FLARE as a transient reporter to obviate the need for generating stable cell lines and allow ‘scarless’ co-selection of genetically engineered cells. We tested this hypothesis by generating point mutations in two genes implicated in oncogenesis: EGFR and BRAF. Specifically, we generated the EGFR T790M mutation, or an observed clinical mutation in BRAF (T57I), or  BRAF knock-out (KO), with simultaneous editing of BE-FLARE. The BRAF mutations were generated using a single gRNA designed to introduce a premature stop codon at position Q58, or a subsitution at T57. In all cases, a non-targeting gRNA served as a negative control. We FACS sorted GFP-positive cells and used Sanger sequencing to quantify editing efficiencies (Fig. 3a). For BRAF and EGFR, GFP-sorted cells had a striking increase in editing compared with transfected cells that were mock-sorted. For BRAF, average editing was increased from ~ 9 to 55% at T57 and from ~ 4 to 20 % at Q58, and for EGFR T790M, from ~ 1 to 18% (Fig. 3b). The lower levels of base editing for EGFR may reflect the increased EGFR allele copy number in PC9 cells [14]. Taken together, these data demonstrate the use of transient expression of BE-FLARE in the enrichment of co-editing events in mammalian cells.

Next, we sought to determine how BE-FLARE would compare with marking BE-transfected cells with a fluorescent reporter (TurboRFP) coded in the BE transcript via a self-cleaving T2A peptide (Fig. 3c). Interestingly, the majority of cells that had undergone base editing of BE-FLARE (i.e. GFP-positive cells) remained RFP-negative (Fig. 3d). Thus, expression of BE3-T2A-RFP below the level detectable by flow cytometry is still functional in cells, implying that using RFP expression for selection would significantly underestimate the number of edited cells. Moreover, the high levels of BE3 expression apparent in the RFP-positive population may not be desirable for many applications due to the possibility of increasing off-target editing.

### Activity measurement of APOBEC-Cas9 fusion variants using BE-FLARE

**Fig. 4:**
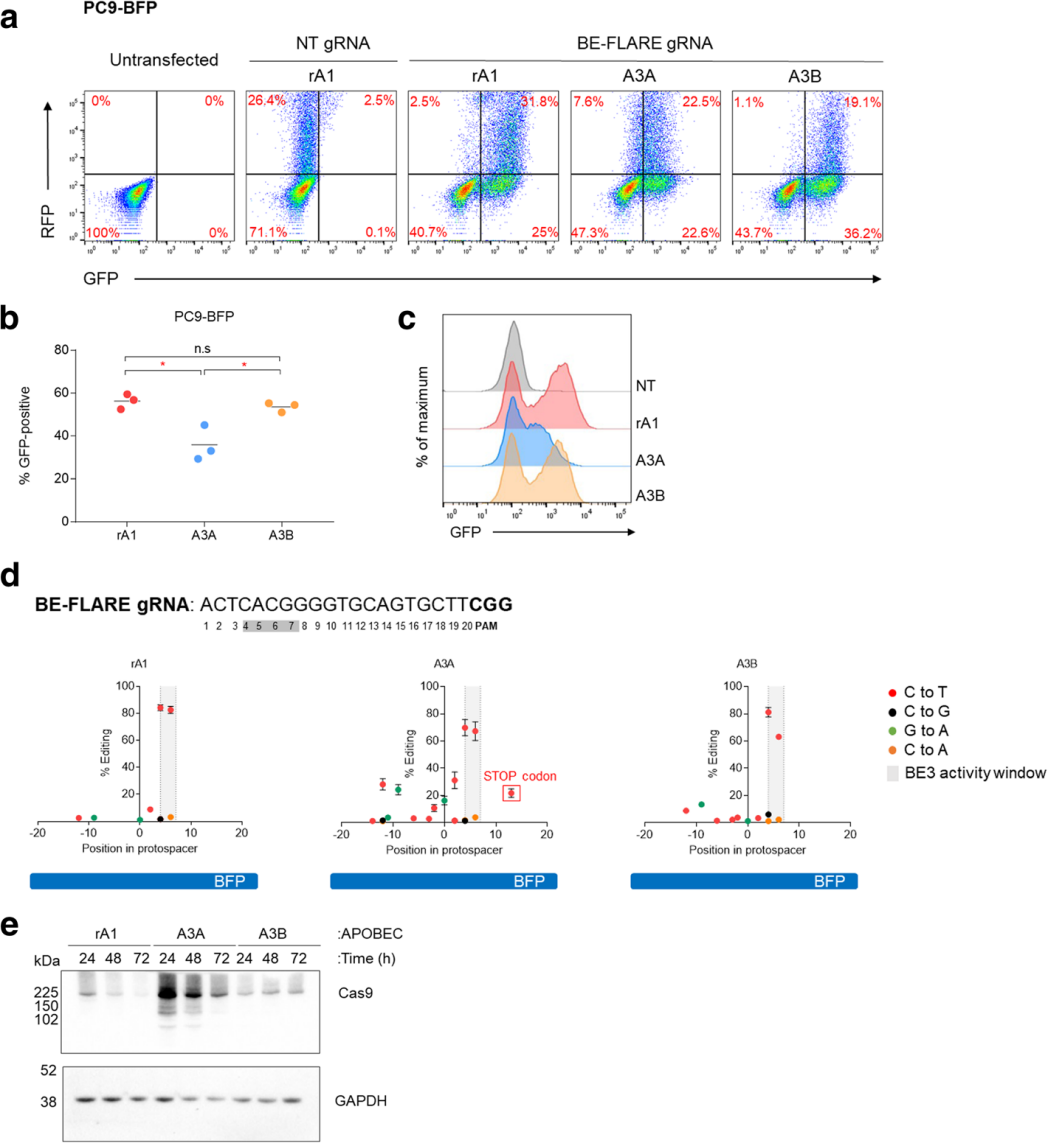
Evaluation of base editor variants with BE-FLARE. **a** Comparison of APOBEC-BE3-T2A-TurboRFP variants with BE-FLARE. PC9 BE-FLARE stable cells were transiently transfected with BE3-T2A-RFP versions where the cytosine deaminase domain of BE3 was rA1, A3A or A3B co-expressing non-targeting gRNA (NT) or BFP gRNA (BFP gRNA). The frequency of GFP-positive cells was quantified by flow cytometry after 72 h. Data are representative of three independent experiments. **b** Quantification of the experiments described in (**a**). Mean ± SD from three independent experiments. Unpaired Student’s *t* test; *P < 0.05. **c** Representative histograms from flow cytometry analysis of GFP fluorescent signal from the experiments described in (**b**). Data are representative of three independent experiments. **d** Base editing of BE-FLARE by rA1-BE3 (left), A3A-BE3 (middle) or A3B-BE3 (right) transfected cells assessed by amplicon sequencing of the BFP locus. GFP-positive cells were FACS sorted 72 h after transfection, and 5 days later, genomic DNA was taken for amplicon sequencing. BE with a non-targeting guide served as a negative control (see Additional file 1: Table S3). Boxed in red is a bystander mutation leading to the introduction of a premature stop codon. Data represent the mean ± SD from two independent experiments and include SNPs found at ≥ 1% of total reads per sample. Read counts can be found in Additional file 1: Table S3. Data from these experiments are also part of Fig. 1c. **e** Immunoblot analysis of PC9 BE-FLARE cells 24, 48 and 72 h post-transfection with the indicated base editor variants (not T2A-RFP). Base editor expression over time was tracked by Cas9 immunodetection. Data are representative of two independent experiments. Raw data can be found in Additional file 2

Several reports have tried using alternative cytidine deaminase domains to drive base editing [2, 3, 15]. We sought to use BE-FLARE to provide a sensitive assessment of alternative domains by monitoring the frequency of BFP to GFP transitions. Specifically, we replaced the original rat APOBEC-1 (rA1) cytidine deaminase domain of BE3 with human APOBEC3A (A3A), or human APOBEC3B (A3B) C-terminal domain. To control for relative transfection efficiency and protein expression levels, we monitored levels of each of the BE versions at the protein level using T2A-TurboRFP (Additional file 1: Figure S3). Interestingly, we found that A3B-BE3 had comparable activity to rA1-BE3 (Fig. 4a and b, Additional file 1: Figure S3), suggesting that A3B may be a tractable alternative to rA1 within BE3. A3A-BE3, however, had significantly lower activity than rA1 or A3B variants (Fig. 4b), and a proportion of cells expressing A3A-BE3 were still GFP-negative (Fig. 4a). Moreover, the resultant GFP-positive cells produced by A3A-BE3 expressed lower levels of GFP than rA1 or A3B fusions (Fig. 4c). Consistent with our results from the transient transfections with BE-FLARE (Fig. 3d), only the minority of cells that had undergone BFP->GFP editing were RFP-positive. The level of BE-FLARE protein expression was only modestly reduced 72 h after transfection, implying that residual BE-FLARE protein and WT BE-FLARE contribute to retention of BFP expression after editing (Additional file 1: Figure S4).

We further analysed the editing pattern of the three APOBEC-BE3 variants by next-generation sequencing of the targeted BFP amplicons in GFP-positive cells isolated by FACS (Fig. 4d). On-target C->T editing within the optimal editing window at codon H66 was higher for rA1 and A3B than A3A. We observed equal editing frequency of the CAC H66 codon for rA1 and A3A but higher stringency observed for A3B, which seems more capable of discriminating between these two proximal cytosine targets within the optimal editing window. We observed similarly low levels of non-C->T mutations, including C->G and C->A variants for all three APOBEC variants. Interestingly, A3A produced more bystander C->T and G->A (on the complementary strand) mutations than either rA1 or A3B versions of BE3. Moreover, we observed low-frequency G->A mutations as far as − 86 (1.3 ± 0.04% allele frequency) and + 76 (1.3 ± 0.1% allele frequency) relative to the protospacer start position, exclusively in the A3A-edited samples (not shown). Notably, one of the bystander mutations produced a premature stop codon (21.8 ± 2.2% allele frequency; Fig. 4d), which likely explains the reduction in GFP expression observed in A3A-BE3 edited cells.

To better understand the potential mechanisms behind the differing mutational characteristics of each APOBEC-BE3 variant, we analysed the protein expression of the base editors (Fig. 4e). A3A-BE3 had the highest expression of all at the time points tested, perhaps explaining the increased levels of undesired bystander mutations. rA1-BE3 levels were comparable to that of A3B-BE3 but decayed more rapidly over time. Indeed, at 72 h post-transfection, rA1-BE3 was undetectable by Western blotting, whereas A3B-BE3 was still at levels comparable to 24 h post-transfection. Taken together, these results suggest that these APOBEC-Cas9 fusions have drastically different protein expression and/or stability in mammalian cells, which may partially explain base editing characteristics such as efficiency and precision. Notably, we used a codon-optimised version of rat APOBEC1 in BE3 throughout this report, as we found that the codon-optimised version was expressed at much higher levels than the native sequence when assessing RFP expression in a BE3-T2A-truboRFP system (Additional file 1: Figure S5), which is consistent with recent reports [16, 17].

**Fig. 5:**
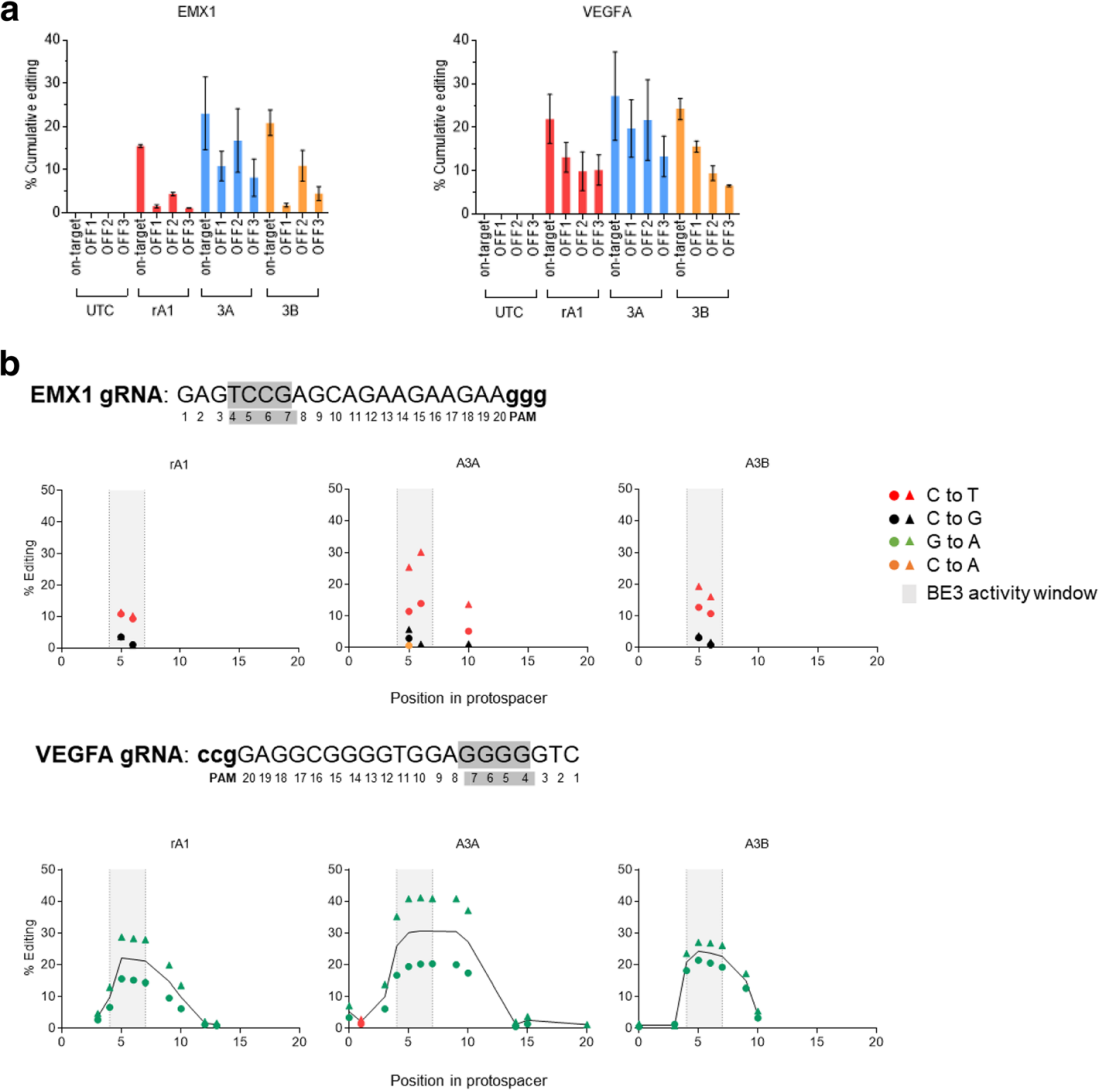
Amplicon sequencing reveals efficient editing by A3A and A3B base editors, but reduced precision with A3A. **a** Amplicon sequencing data for *EMX1* and *VEGFA* editing in HEK293 cells 48 h post-transfection, expressed as percentage cumulative editing of the indicated locus. Data represent the mean ± SD from two independent experiments and include SNPs found at ≥ 1% of total reads per sample. UTC, untransfected control. All read counts can be found in Additional file 1: Table S4 and Table S5. **b** Amplicon sequencing data for *EMX1* and *VEGFA* editing in HEK293 cells 48 h post-transfection, expressed as percentage nucleotide substitution at that position in the protospacer. The optimal base editing activity window is highlighted in grey. Data are from two independent experiments (circles, exp.1; triangles, exp.2) and include SNPs found at ≥ 1% of total reads per sample. For *VEGFA*, a connecting line joins the mean % editing. Untransfected cells served as a control. All read counts can be found in Additional file 1: Table S4 and Table S5. Raw data can be found in Additional file 2

To further analyse the editing profile of A3A and A3B base editor variants, we set out to analyse on-target and off-target editing by next-generation sequencing of PCR amplicons. Using HEK293 cells, we targeted *EMX1* and *VEGFA* loci and measured on-target editing, three previously reported off-target sites for each gRNA (Additional file 1: Table S4 and Table S5), and bystander mutations within the gRNA binding site. We found on-target editing of *EMX1* and *VEGFA* to be modestly higher for A3A when compared with rA1 and A3B base editor variants; however, A3A-BE3 generally produced higher frequencies of off-target events within 6/6 of the off-target loci tested (Fig. 5a). Furthermore, when analysing the profile of on-target editing of *EMX1* and *VEGFA* loci, we noted an increased frequency of bystander C->T and G->A editing events in the A3A-BE3 samples, implying a broader editing window within the gRNA binding sequence (Fig. 5b). For example, only A3A-BE3 produced bystander mutations at position + 10 in the *EMX1* protospacer and beyond position + 13 in the *VEGFA* protospacer. In contrast, rA1-BE3 and A3B-BE3 displayed narrow activity windows within the protospacer sequence, with comparable on-target editing efficiencies.

In conclusion, our deep sequencing analyses are broadly consistent with results generated from editing BE-FLARE. We demonstrate that A3A-BE3 has a broader mutational profile leading to higher bystander mutation rates which is consistent with loss of GFP fluorescence in the BE-FLARE system. Thus seen, BE-FLARE is a valuable tool for measuring the efficiency and precision of novel base editors.

## Discussion

We employed BE-FLARE to evaluate the efficiency and precision of different base editor variants. Replacement of rat APOBEC1 in BE3 with human APOBEC3B resulted in a similar level of activity and specificity as the original, codon-optimised rat APOBEC1. Surprisingly, A3A-BE3 induced greater bystander mutations, which we further confirmed at multiple genomic on-target and off-target sites. Although A3A and A3B C-terminal domain share ~ 90% similarity in protein sequence, the active site in A3A is open whereas in A3B it is partially occluded by the flexible loop 1 region (Additional file 1: Figure S6) [18–20]. Whilst these structural differences could explain the increased bystander rates of A3A-BE3, we also observed a significant increase in protein expression over time, which suggests there could be a fine balance between protein abundance/stability and precision of base editors. This is supported by the recent finding that codon optimisation of BE3 constructs significantly increase activity [16, 17], suggesting that protein expression from the original BE3 constructs is a limiting factor. Whilst undesirable for precision applications, the increased bystander editing frequency of A3A-BE3 may prove beneficial for targeted mutagenesis approaches similar to the CRISPR-X system [21, 22]. A recent study demonstrated that several point mutations in A3A can reduce bystander mutation rates of A3A-BE3 [15], showing that this highly active cytidine deaminase can be rationally refined for gene editing. Our findings imply that A3B may also prove to be an excellent starting point to develop more precise base editors with increased editing efficiency, whilst the reduction of immunogenic peptides compared to rA1 could be beneficial in therapeutic settings.

We have developed a capability to enrich genetically edited cells through selection based upon a fluorescent reporter of base editing activity. Transient expression and selection of BE-FLARE-positive cells allowed significant enrichment of base editing at secondary sites. This methodology is broadly applicable and can help when generating a modified cell line by reducing the number of clones screened to identify the desired genotype, especially when no phenotypic selection is possible or where the desired mutation has deleterious effects on cell fitness. The importance of this benefit is highlighted by the low level of base editing of EGFR observed in mock-selected pools compared to the significant increase after BE-FLARE enrichment. This low level of editing is likely a result of the high EGFR copy number in PC9 cells, which are dependent upon mutant EGFR signalling for survival [13]. As gene copy number alterations are common in cancer cell lines, enrichment before generation of single cell clones offers an invaluable tool to improve the success of cell model generation. At certain sites, BE-FLARE enrichment generated very high levels of editing in bulk pools, which in some cases may avoid the need to use single-cell clones entirely.

Alternative BE reporter systems have been recently described; a fluorescent Stop-GFP reporter, where a stop codon is mutated in order to activate GFP expression, was used to monitor activity of Cas9 fused to activation-induced cytosine deaminase [21, 23]. This reporter lacks a detectable signal before editing, making it difficult to monitor the efficiency of reporter delivery, or infer ratios of edited versus unedited reporter. Another study described a reporter which required a dual guide approach to generate two proximal uracil—ssDNA nicks, repair of which can lead to in-frame InDel events and restoration of the mCherry reading frame [24]. The authors achieved enrichment of edited cells by sorting mCherry-positive cells, but how sensitive InDel formation is as a measure of base editor activity is unclear. In contrast, BE-FLARE provides a direct read-out on desired point mutation events, which is the prevailing product of base editing [1]. Finally, a similar BFP to GFP strategy has been reported to detect base editing events in plant cells [25]; our complementary data in human cells represent the first instance of its use in selection for co-editing events. A current limitation of our reporter strategy is that it provides a relative measure of BE/cytidine deaminase activity rather than an absolute measure, since a small number of editing events may result in InDels or perfect DNA repair, both of which will not give rise to GFP-expressing cells. We envisage that BE-FLARE could be further refined to contain a single cytosine in the histidine 66 codon to allow for easy reversal of editing back to the WT BFP sequence using the recently published A->G Adenosine base editor (ABE) [26]. This would allow for tracking of cells that have undergone a transition from BFP to GFP and then back to BFP, facilitating genetic rescue experiments on endogenous genes.

Using an alternative approach, we marked transfected cells with a co-expressed fluorescent protein. Whilst this system is suitable for transient transfection, the large size of the final expression cassettes at nearly 8.5 kb precludes such delivery by lentivirus, where cargo sizes are limited [27]. Importantly, BE-FLARE directly reports on base editor activity rather than simply the expression of BE3, providing a more functional read-out. Separate delivery of BE-FLARE allows for maximum flexibility and applicability in multiple cell systems and with various base editor versions. Finally, cell lines stably expressing the BE-FLARE allow for tracking of edited cells over time to monitor phenotype, which is not possible with transient expression of a fluorescent protein. Indeed, as GFP fluorescence is amenable to detection by fluorescence microscopy, BE-FLARE can be applied to detect base editing in high-throughput functional genetic screens. This reporter may also be employed in therapeutic genome editing, where it is important to select for rare editing events in primary cells without introducing a permanent genetic marker.

## Conclusions

In conclusion, we present BE-FLARE as a rapidly implementable system for tracking and selecting base edited cells and refining the next generation of base editors.

## Methods

### Cell culture

HEK 293 and PC9 (both from ATCC) cells were maintained at 5% CO_2_, 95% air in RPMI, 10% FCS, 1 X GlutaMAX (ThermoFisher). Transfections were performed using FuGENE HD (Promega) using a 3:1 ratio of transfection reagent to DNA according to the manufacturer’s instructions. Cell lines were STR profiled and verified as mycoplasma-free.

### Cloning and plasmids

The BE3 expression cassette was synthesised (ThermoFisher) and cloned into pcDNA3.1(+). We introduced a cassette into the Mlu site containing an AarI guide cloning region with ccdB for selection, and the human U6 promoter driving gRNA expression. gRNA sequences were cloned into the AarI site using complementary primer pairs, which were annealed, phosphorylated, and ligated into the linearised vector. Primers can be found in Additional file 1: Table S1. For the BFP reporter construct, a gBlock encoding eBFP was synthesised (IDT) and introduced by Gibson assembly (NEB) into an expression vector under the human EF-1 alpha promoter. The vector contains sequences to allow ObLiGaRe-mediated integration into the human AAVS1 ‘safe harbour’ locus [11]. All sequences of the synthesised cassettes and guide RNAs are listed in Additional file 1: Supplementary Methods. *VEGFA*, *EMX1* and non-targeting guide RNAs are published [15, 28, 29].

### Generation of stable BE-FLARE cell lines

HEK293 and PC9 cells were transfected with BE-FLARE plasmid and a construct encoding zinc-fingers targeting the AAVS1 safe-harbour locus, essentially as described [11], and subsequently selected for 3 days with puromycin (1 μg/ml).

### Flow cytometry

FACS was carried out on a FACSJazz (BD Biosciences), and flow cytometry analysis was carried out on a Fortessa (BD Biosciences). Briefly, cells were transfected with the indicated constructs and, 3 days later, harvested by trypsinisation for flow cytometry analysis or FACS.

### Next-generation sequencing

Forty-eight hours after transfection with the indicated BE3 variant (1 μg of plasmid per well of 12-well plate with FuGene HD; Promega), genomic DNA was generated from the resultant pool of HEK293 cells using DNA Blood/Tissue Kit (Qiagen). PCR1 amplicons were generated using primers containing adapter sequences as stated in Additional file 1: Table S2. PCR1 primers for human (HEK293 cell) *EMX1* [30], *VEGFA* [15], and *VEGFA* off-targets [28] are published. Genomic DNA was amplified based on the predetermined minimal PCR cycle number required, which ranged between 22 and 25 cycles. Indexing primers were added in a second PCR step with a further 10 PCR cycles using 1 ng of purified PCR product from PCR1. For all PCR reactions, amplicons were cleaned-up using MAGBIO magnetic SPRI beads and amplicon size was validated using the QIAxcel (QIAGEN). Libraries were quantified using KapaQuant qPCR kit (KAPA Biosystems), pooled and sequenced on a MiSeq (Illumina).

### Bioinformatics

Base editing efficiencies were estimated from Sanger sequence chromatograms using EditR [31], or by analysis of NGS. For amplicon sequencing data analyses, Fast Length Adjustment of Short reads (FLASH v1.2.11) was used to group paired reads. BWA-MEM was used to align to the human genome (hg19) or the BFP coding sequence. Samtools was used to generate sorted, indexed BAM files. Samtools was used to generate data for variant calling with the following options: minimum read depth 50, minimum quality 25, minimum allele frequency 0.005, maximum mismatch 100, and trim 20 [32].

### Western blotting

Whole cell lysates were generated using RIPA buffer (ThermoFisher Scientific), and Western blotting was performed using standard methods, with secondary antibodies conjugated to horseradish peroxidase (GE Healthcare). Cas9 (#14697; RRID: AB_2750916) and GAPDH (#2118; RRID: AB_561053) antibodies were from Cell Signaling Technology.

### Digital droplet PCR (ddPCR)

Base editing over time was estimated by extraction of genomic DNA with DNAeasy Blood & Tissue kit (Qiagen) followed by ddPCR with ddPCR Supermix for probes no dUTP (BioRad) according to the manufacturer’s instructions. Probes were labelled with FAM and are listed in Additional file 1: Supplementary Methods.

### Experimental design and statistics

The exact value of sample size (*n*), statistical tests used, and the number of independent experiments performed are given in the figure legends. Unless otherwise stated, error bars represent standard deviation and an unpaired Student’s *t* test was used to assess statistical significance (*P* < 0.05).

## Additional files

Additional file 1: **Figure S1–S6, Table S1–S5,** Supplementary methods. **Figure S1.** BE-FLARE facilitates visual tracking of base edited cells by microscopy. **Figure S2.** BE-FLARE base editing over time reveals dynamics of base editing and tracking of edited cells with GFP. **Figure S3.** Raw data relating to Fig. 4a b and c, and quantification of turbo-RFP positive cells. **Figure S4.** BE-FLARE expression after editing. **Figure S5.** Expression of native vs codon-optimised rat APOBEC-1 BE3 reveals superior expression after codon optimisation. **Figure S6.** Sequence alignment of APOBECs highlights divergent loop1 region. Supplementary methods. **Table S1.** Primers for guide RNA cloning. **Table S2.** Primers for amplicon sequencing. **Table S3.** Amplicon sequencing summary: BE-FLARE (BFP). **Table S4.** Amplicon sequencing summary: *EMX1*. **Table S5.** Amplicon sequencing summary: *VEGFA*. Digital droplet PCR probes. Sequences of constructs. (DOCX 1133 kb)

Additional file 2: Raw data relating to Figs. 1, 2, 3, 4 and 5, Figure S2, S3, S4 and S5. (XLSX 34 kb)
