# Supplementary material for: BE-FLARE: a fluorescent reporter of base editing activity reveals editing characteristics of APOBEC3A and APOBEC3B
Source: BMC Biol. 2018 Dec 28;16:150. doi: 10.1186/s12915-018-0617-1 (PMC6309101; doi:10.1186/s12915-018-0617-1)
Supplement: Supplementary file 1 — Figure S1–S6, Table S1–S5, Supplementary methods. Figure S1. BE-FLARE facilitates visual tracking of base edited cells by microscopy. Figure S2. BE-FLARE base editing over time reveals dynamics of base editing and tracking of edited cells with GFP. Figure S3. Raw data relating to Fig. 4a b and c, and quantification of turbo-RFP positive cells. Figure S4. BE-FLARE expression after editing. Figure S5. Expression of native vs codon-optimised rat APOBEC-1 BE3 reveals superior expression after codon optimisation. Figure S6. Sequence alignment of APOBECs highlights divergent loop1 region. Supplementary methods. Table S1. Primers for guide RNA cloning. Table S2. Primers for amplicon sequencing. Table S3. Amplicon sequencing summary: BE-FLARE (BFP). Table S4. Amplicon sequencing summary: EMX1. Table S5. Amplicon sequencing summary: VEGFA. Digital droplet PCR probes. Sequences of constructs. (DOCX 1133 kb) [file 12915_2018_617_MOESM1_ESM.docx]

**Additional file1: Supplementary Figures and Methods**

**Supplementary Figures**

**
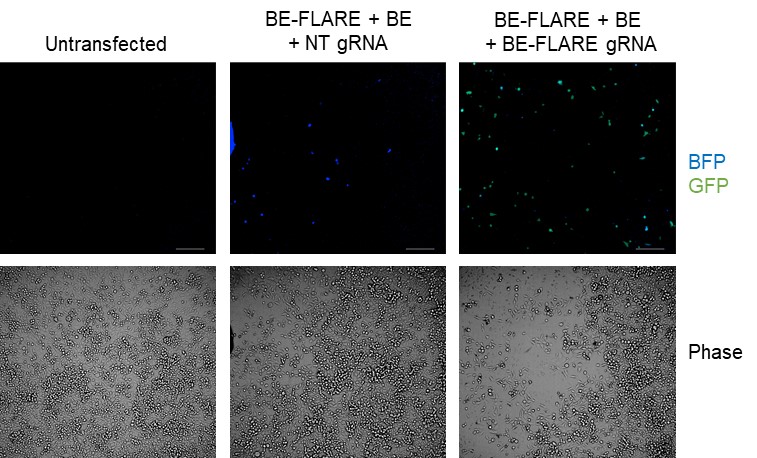
**

**Figure S1. BE-FLARE facilitates visual tracking of base edited cells by microscopy.**

PC9 cells were cotransfected with plasmids encoding BE-FLARE, BE3 and a guide RNA targeting BFP codon H66. 72 h later, BFP and GFP fluorescence was acquired by confocal microscopy. Edited cells are GFP-positive, non-edited cells are BFP-positive. Data are representative of two independent experiments. Scale bar denotes 200 um.


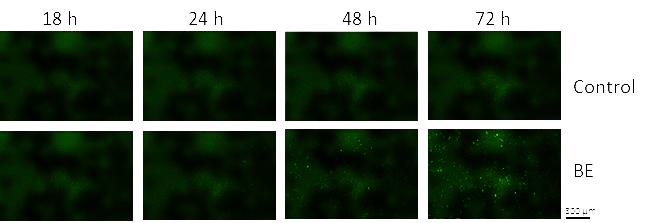


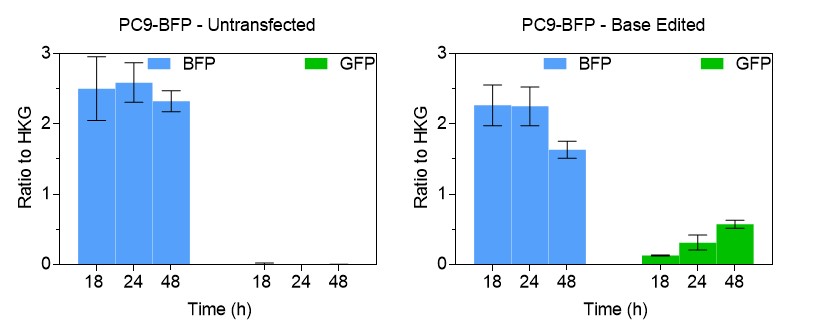


**Figure S2. BE-FLARE base editing over time reveals dynamics of base editing and tracking of edited cells with GFP.**

PC9-BE-FLARE cells were transfected with a plasmid encoding BE3 and a guide RNA targeting BFP codon H66. GFP images were acquired on the Incucyte. Edited cells are GFP-positive. Data are representative of two independent experiments.

For digital droplet PCR (ddPCR), genomic DNA was extracted at the indicated time points and editing was monitored using ddPCR with custom probes targeting WT or edited BE-FLARE (Supplementary Methods). Data are represented as the mean ± SD of two independent experiments.


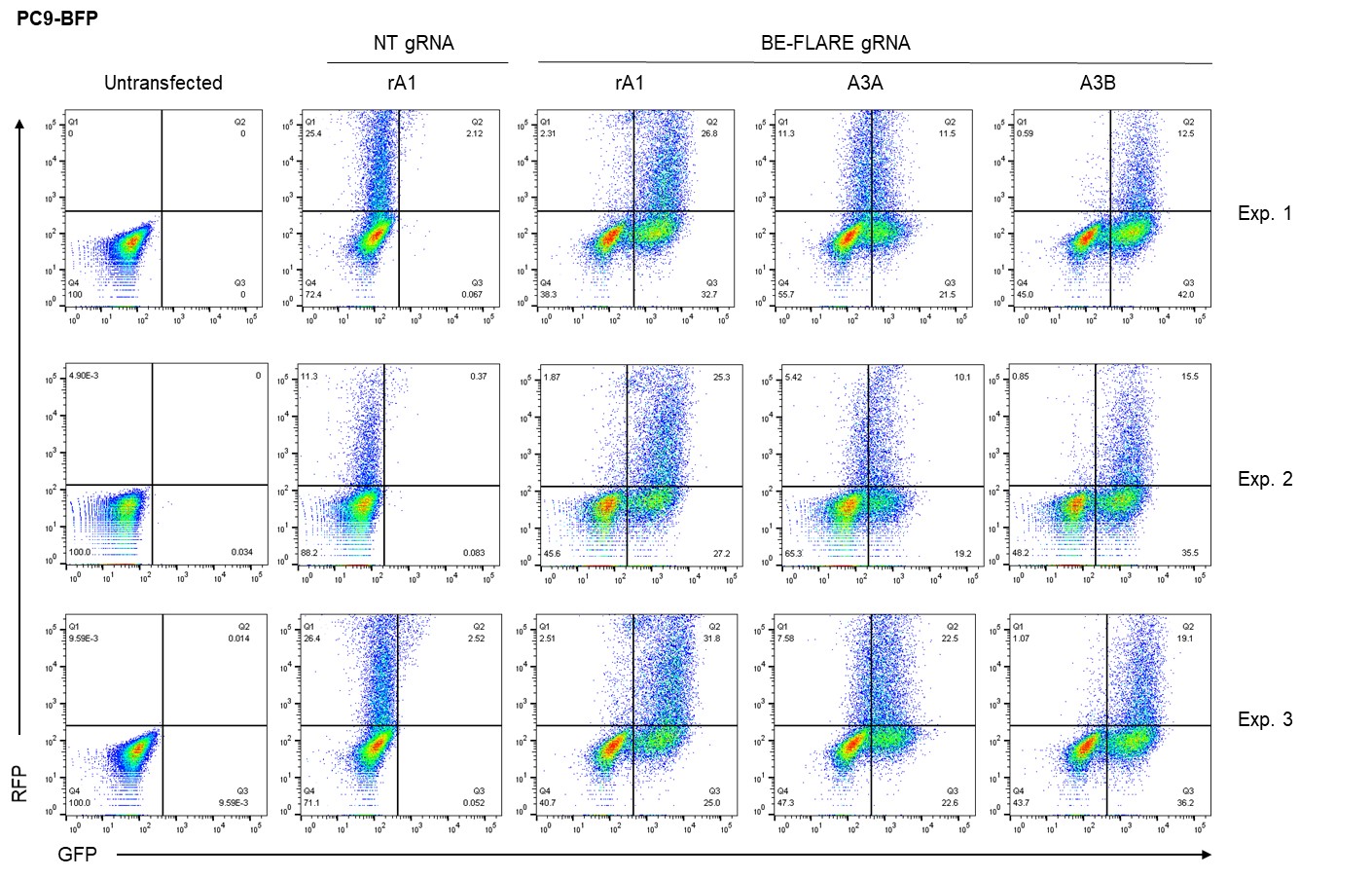


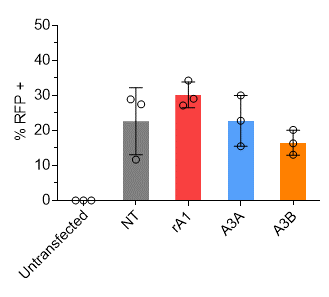


**Figure S3. Raw data relating to Figure 4a, 4b and 4c and quantification of turbo-RFP positive cells.**

PC9-BE-FLARE cells were transfected with the indicated BE3-variants targeting BE-FLARE with BE-FLARE gRNA. 72 h later, cells were harvested for flow cytometry analysis to monitor GFP and RFP positive cells. All three independent experiments relating to Figure 4a, 4b and 4c are shown, including summary data of RFP events in each case (mean ± SD of three independent experiments).


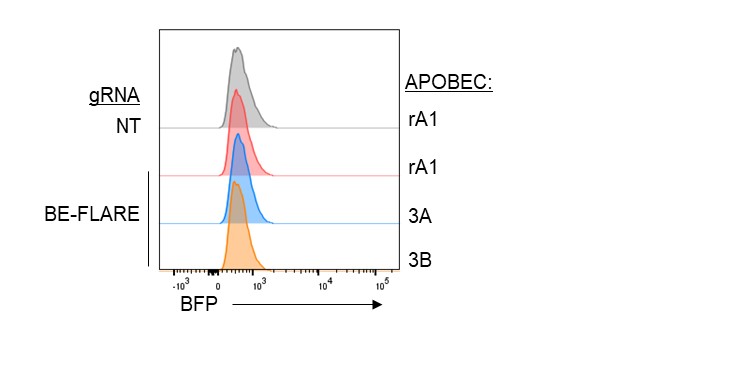


**Figure S4. BE-FLARE expression after editing.**

PC9-BE-FLARE cells were transfected with rA1, A3A and A3B BE3 variants targeting BE-FLARE with BE-FLARE gRNA. 72 h later, BFP expression was monitored by flow cytometry to detect changes in BE-FLARE expression after editing. Data are representative of three independent experiments.


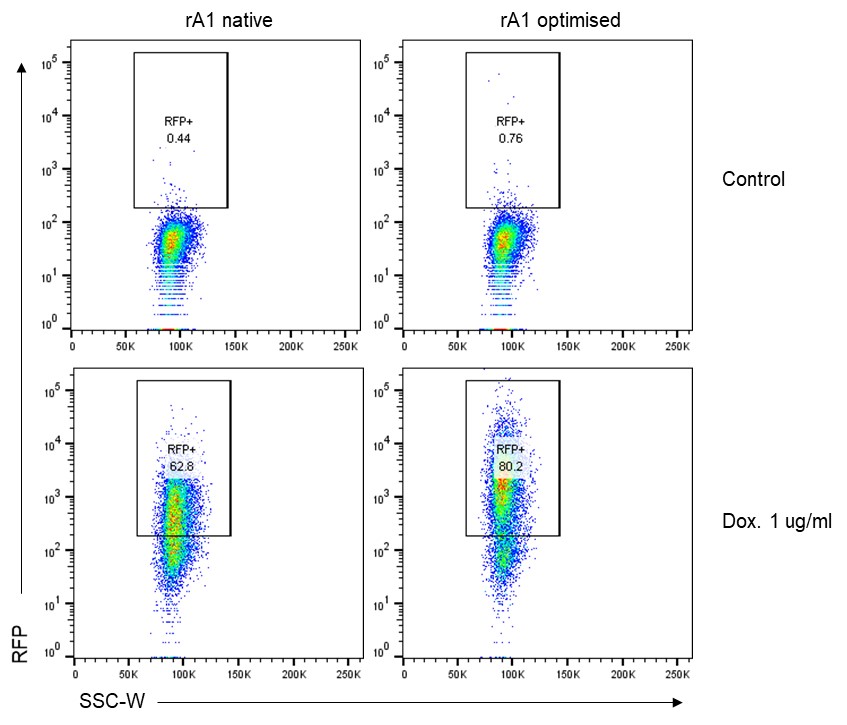


**Figure S5. Expression of native vs codon-optimised rat APOBEC-1 BE3 reveals superior expression after codon optimisation.**

HEK293 cells were engineered to stably express a doxycycline-inducible version of BE3-T2A-turboRFP. Native and codon-optimised rat APOBEC1-BE versions were tested. After addition of doxycycline for 72, cells were harvested for flow cytometry analysis of turboRFP expression. Data are representative of two independent experiments.


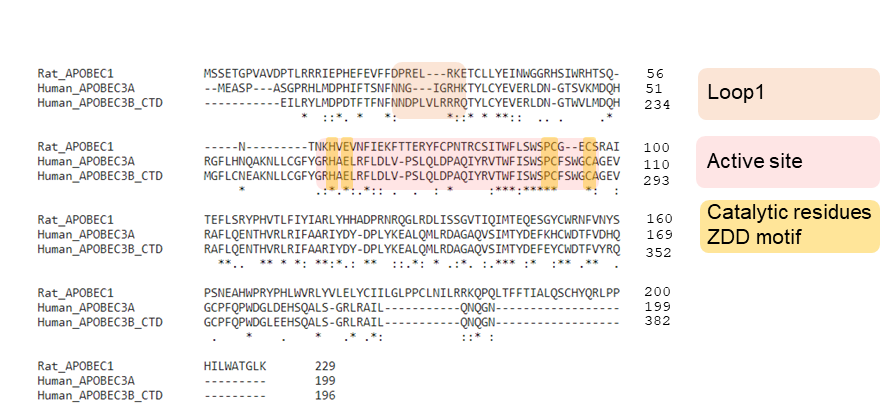


**Figure S6. Sequence alignment of APOBECs highlights divergent loop1 region.**

Clustal Omega sequence alignment of the three APOBEC proteins used in this study. Indicated are the divergent loop1 region, the zinc-coordinating active site region including key catalytic residues in the zinc-dependent deaminase signature motif.

**Supplementary Methods**

**Table S1. Primers for guide RNA cloning**

|  | **Forward primer** | **Reverse primer** |
| --- | --- | --- |
| **Non-targeting (NT) guide RNA** | **ACCG**GCCCCGCCGCCCTCCCCTCC | **AAAC**GGAGGGGAGGGCGGCGGGGC |
| **BE-FLARE guide RNA** | **ACCG**ACTCACGGGGTGCAGTGCTT | **AAAC**AAGCACTGCACCCCGTGAGT |
| **EGFR T790M guide RNA** | **ACCG**ATCACGCAGCTCATGCCCTT | **AAAC**AAGGGCATGAGCTGCGTGAT |
| **BRAF T57I/Q58 guide RNA** | **ACCG**GTTGACACAGGAACATATAG | **AAAC**CTATATGTTCCTGTGTCAAC |
| **EMX1 guide RNA** | **AACG**GAGTCCGAGCAGAAGAAGAA | **AAAC**TTCTTCTTCTGCTCGGACTC |
| **VEGFA guide RNA** | **AACG**GACCCCCTCCACCCCGCCTC | **AAAC**GAGGCGGGGTGGAGGGGGTC |

Overhangs required for ligation into the AarI site are in bold.

**Table S2. Primers for amplicon sequencing**

|  | **Forward primer** |
| --- | --- |
| **EMX1** | **TCGTCGGCAGCGTCAGATGTGTATAAGAGACAG**GGACAAAGTACAAACGGCAGAAGCTGG |
| **EMX1 OFF 1** | **TCGTCGGCAGCGTCAGATGTGTATAAGAGACAG**TTCTGAGGGCTGCTACCTGT |
| **EMX1 OFF 2** | **TCGTCGGCAGCGTCAGATGTGTATAAGAGACAG**CACGGCCTTTGCAAATAGAG |
| **EMX1 OFF 3** | **TCGTCGGCAGCGTCAGATGTGTATAAGAGACAG**CCAGACTCAGTAAAGCCTGGA |
| **VEGFA** | **TCGTCGGCAGCGTCAGATGTGTATAAGAGACAG**CTGACCAGTCGCGCTGACGG |
| **VEGFA OFF 1** | **TCGTCGGCAGCGTCAGATGTGTATAAGAGACAG**TCCTACAAGTAACAGTCCAAGAA |
| **VEGFA OFF 2** | **TCGTCGGCAGCGTCAGATGTGTATAAGAGACAG**ACCAAGCCCATTTGTCCAGG |
| **VEGFA OFF 3** | **TCGTCGGCAGCGTCAGATGTGTATAAGAGACAG**TCCATACCAGCAGCAGTTCC |
| **BE-FLARE** | **TCGTCGGCAGCGTCAGATGTGTATAAGAGACAG**TCAGCGTGTCCGGCGAGGGC |
|  | **Reverse primer** |
| **EMX1** | **GTCTCGTGGGCTCGGAGATGTGTATAAGAGACAG**GAGTGGCCAGAGTCCAGCTTGG |
| **EMX1 OFF 1** | **GTCTCGTGGGCTCGGAGATGTGTATAAGAGACAG**GCCCAATCATTGATGCTTTT |
| **EMX1 OFF 2** | **GTCTCGTGGGCTCGGAGATGTGTATAAGAGACAG**GGCTTTCACAAGGATGCAGT |
| **EMX1 OFF 3** | **GTCTCGTGGGCTCGGAGATGTGTATAAGAGACAG**TGGCCCCAGTCTCTCTTCTA |
| **VEGFA** | **GTCTCGTGGGCTCGGAGATGTGTATAAGAGACAG**CAGAAGTTGGACGAAAAGTTTCAGTGCG |
| **VEGFA OFF 1** | **GTCTCGTGGGCTCGGAGATGTGTATAAGAGACAG**TTCTGCAACTTAACTTACGTGAAA |
| **VEGFA OFF 2** | **GTCTCGTGGGCTCGGAGATGTGTATAAGAGACAG**TCCTTCTTTTTGAGCTTTGGGC |
| **VEGFA OFF 3** | **GTCTCGTGGGCTCGGAGATGTGTATAAGAGACAG**CTCACCTCAGCTCCTGCAC |
| **BE-FLARE** | **GTCTCGTGGGCTCGGAGATGTGTATAAGAGACAG**TGTCGCCCTCGAACTTCACC |

Adapter sequences for binding indexing primers are in bold.

**Table S3. Amplicon sequencing summary: BE-FLARE (BFP)**

| **BE3 deaminase:** | **Guide RNA** | **# reads with SNPs exp.1** | **# reads with SNPs exp.2** |  |
| --- | --- | --- | --- | --- |
| ratA1 | NT | 0 (0/863842) | 0 (0/974555) |  |
| ratA1 | BFP | 641341 | 770475 |  |
| APOBEC 3A | BFP | 376382 | 536436 |  |
| APOBEC 3B | BFP | 582945 | 765190 |  |
|  |  |  |  |  |
|  |  |  |  |  |

**Table S4. Amplicon sequencing summary: *EMX1***

Mismatches in the guide RNA binding site and PAM sequence are in red.

**Table S5. Amplicon sequencing summary: *VEGFA***

Mismatches in the guide RNA binding site and PAM sequence are in red.

**Digital Droplet PCR probes for BE-FLARE**

FAM_WT: CTG ACT {C}A{C} GGG GTG C

FAM_Uracil: CTG CAC CCC {A}T{A} AGT CAG GGT

{ } denotes Locked Nucleic Acid (LNA) positions.

Note that the FAM_Uracil probe is complementary to BE-FLARE and is designed to detect U or T editing of codon 66.

**Modified enhanced BFP (eBFP) sequence to report on base editing activity: BE-FLARE**

atggtgagcaagggcgaggagctgttcaccggggtggtgcccatcctggtcgagctggacggcgacgtaaacggccacaagttcagcgtgtccggcgagggcgagggcgatgccacctacggcaagctgaccctgaagttcatctgcaccaccggcaagctgcccgtgccctggcccaccctcgtgaccaccctgactcacggggtgcagtgctt**cgg**tcggtaccccgaccacatgaagcagcacgacttcttcaagtccgccatgcccgaaggctacgtccaggagcgcaccatcttcttcaaggacgacggcaactacaagacccgcgccgaggtgaagttcgagggcgacaccctggtgaaccgcatcgagctgaagggcatcgacttcaaggaggacggcaacatcctggggcacaagctggagtacaactacaacagccacaacgtctatatcatggccgacaagcagaagaacggcatcaaggtgaacttcaagatccgccacaacatcgaggacggcagcgtgcagctcgccgaccactaccagcagaacacccccatcggcgacggccccgtgctgctgcccgacaaccactacctgagcacccagtccgccctgagcaaagaccccaacgagaagcgcgatcacatggtcctgctggagttcgtgaccgccgccgggatcactctcggcatggacgagctgtacaagtaa

The protospacer is underlined. The PAM is in bold.

Sequence of NLS-**ratA1**-XTEN linker-Cas9(D10A)-UGI-T2A-TurboRFP BE3

atggctcctaagaaaaagcggaaggtg**tctagcgagacaggccctgtggccgtggatcctacactgcggagaagaatcgagccccacgagttcgaggtgttcttcgaccccagagagctgcggaaagagacatgcctgctgtacgagatcaactggggcggcagacactctatctggcggcacacaagccagaacaccaacaagcacgtggaagtgaactttatcgagaagtttacgaccgagcggtacttctgccccaacaccagatgcagcatcacctggtttctgagctggtccccttgcggcgagtgcagcagagccatcaccgagtttctgtccagatatccccacgtgaccctgttcatctatatcgcccggctgtaccaccacgccgatcctagaaatagacagggactgcgcgacctgatcagcagcggagtgaccatccagatcatgaccgagcaagagagcggctactgctggcggaacttcgtgaactacagccccagcaacgaagcccactggcctagatatcctcacctgtgggtccgactgtacgtgctggaactgtactgcatcatcctgggcctgcctccatgcctgaacatcctgagaagaaagcagcctcagctgaccttcttcacaatcgccctgcagagctgccactaccagagactgcctccacacatcctgtgggccaccggactgaag**tctggctctgagacacctggcacaagcgagtctgccacacctgagtctgacaagaaatactcaatcgggctggccatcggaactaactcagtggggtgggcagtcattactgacgagtacaaagtgccaagcaagaaatttaaggtcctgggcaacaccgataggcactccatcaagaaaaatctgattggggccctgctgttcgactctggagagacagctgaagcaactagactgaaaaggactgctagaaggcgctatacccggcgaaagaatcgcatctgctacctgcaggagattttctctaacgaaatggccaaggtggacgatagtttctttcatcggctggaggaatcattcctggtcgaggaagataagaaacacgagagacatcctatctttggaaacattgtggacgaggtcgcttatcacgaaaaataccccaccatctatcatctgcgcaagaaactggtggactctacagataaagcagacctgcggctgatctatctggccctggctcacatgattaagttcagaggccattttctgatcgagggagatctgaacccagacaatagcgatgtggacaagctgttcatccagctggtccagacatacaatcagctgtttgaggaaaaccctattaatgcatctggcgtggacgcaaaagccatcctgagtgccaggctgtctaagagtagaaggctggagaacctgatcgctcagctgccaggcgaaaagaaaaacggcctgtttggaaatctgattgcactgtcactgggactgacacctaacttcaagagcaattttgatctggccgaggacgctaaactgcagctgagcaaggacacttatgacgatgacctggataacctgctggctcagatcggagatcagtacgcagacctgttcctggccgctaagaatctgtctgacgctatcctgctgagtgatattctgcgggtgaacaccgagattacaaaagcccctctgtcagctagcatgatcaagagatatgacgagcaccatcaggatctgaccctgctgaaggcactggtgcgccagcagctgcccgagaagtacaaggaaatcttctttgatcagagtaagaacgggtacgccggttatattgacggcggagcttcacaggaggaattctacaagtttatcaaacctattctggagaagatggacggcaccgaggaactgctggtgaaactgaatcgcgaggacctgctgcgcaagcagcggacatttgataacggctccatcccccaccagattcatctgggagagctgcacgcaatcctgcgacgacaggaagacttctacccatttctgaaggataaccgcgagaagatcgaaaaaattctgaccttccggatcccttactatgtggggcccctggcaaggggtaattcccgctttgcctggatgacacggaaatctgaggaaacaatcactccttggaacttcgaggaagtggtcgataagggagcttccgcacagtctttcatcgagagaatgacaaacttcgacaaaaacctgccaaatgagaaagtgctgcctaagcacagtctgctgtacgagtatttcacagtctataacgaactgactaaggtgaaatacgtcaccgaggggatgaggaagcccgccttcctgagcggtgaacagaagaaagctatcgtggacctgctgtttaaaaccaatcgcaaggtgacagtcaagcagctgaaggaggactacttcaagaaaattgaatgtttcgattctgtggagatcagtggcgtcgaagacagatttaacgcttctctgggaacctaccacgatctgctgaagatcattaaggataaagacttcctggacaacgaggaaaatgaggatatcctggaagacattgtgctgaccctgacactgtttgaggatcgcgaaatgatcgaggaacggctgaaaacttatgcccatctgttcgatgacaaggtgatgaaacagctgaagcgaagaaggtacaccggctggggacgactgagcagaaagctgatcaacggcattcgggacaaacagagtggaaagactatcctggactttctgaaatcagatggcttcgctaacagaaattttatgcagctgattcacgatgacagcctgaccttcaaagaggatatccagaaggcacaggtgtccgggcagggtgactctctgcacgagcatatcgcaaacctggccgggtcccccgccatcaagaaaggtattctgcagaccgtgaaggtggtcgatgagctggtgaaagtcatgggcaggcataagccagaaaacatcgtgattgagatggcccgcgaaaatcagaccacacagaaaggacagaagaacagccgcgagcggatgaaaaggatcgaggaaggcattaaggaactgggatcccagatcctgaaagagcaccctgtggaaaacactcagctgcagaatgagaagctgtatctgtactatctgcagaatgggcgggatatgtacgtggaccaggagctggatattaaccgactgtctgattacgacgtggatcatatcgtcccacagtcattcctgaaagatgacagcattgacaataaggtgctgacccggagtgacaaaaaccgaggaaagagtgataatgtcccttcagaggaagtggtcaagaaaatgaagaactactggagacagctgctgaatgccaaactgatcacacagcgaaagtttgataacctgactaaagctgagagagggggtctgtcagaactggacaaagcaggcttcatcaagcgacagctggtggagaccagacagatcacaaagcacgtcgctcagattctggatagcaggatgaacacaaagtacgatgagaatgacaaactgatccgcgaagtgaaggtcattactctgaagtcaaaacttgtgagcgacttcagaaaggatttccagttctacaaagtcagggagatcaacaattatcaccatgctcatgacgcatacctgaacgcagtggtcgggaccgccctgattaagaaataccccaaactggagagcgaattcgtgtacggtgactataaggtgtacgatgtcagaaaaatgatcgccaagagtgagcaggaaattggaaaagccaccgctaagtatttcttttactcaaacatcatgaatttctttaagactgagatcaccctggcaaatggggaaatccgaaagagaccactgattgagactaacggcgagaccggagaaatcgtgtgggacaagggtagggattttgccacagtgcgcaaggtcctgtccatgcctcaagtgaatattgtcaagaaaacagaggtgcagactggcggattcagtaaggaatcaattctgcccaaacggaactctgataagctgatcgcccgaaagaaagactgggatcccaagaaatatgggggtttcgactccccaacagtggcttactctgtcctggtggtcgcaaaggtggagaaggggaaaagcaagaaactgaaatccgtcaaggagctgctgggtatcactattatggagaggagctccttcgagaagaaccccatcgattttctggaggctaaaggctataaggaagtgaagaaagacctgatcattaaactgccaaagtacagcctgtttgagctggaaaacggaaggaagcgaatgctggcatccgcaggagagctgcagaagggtaatgaactggccctgccttctaagtacgtgaacttcctgtatctggctagccactacgagaagctgaaaggctcccccgaggataacgaacagaaacagctgtttgtggagcagcacaagcattatctggacgagatcattgaacagattagcgagttctccaaaagagtgatcctggctgacgcaaatctggataaggtcctgagcgcatacaacaaacacagagataagccaatcagggagcaggccgaaaatatcattcatctgttcactctgaccaacctgggagcccctgcagccttcaagtattttgacactaccatcgatcggaaacgatacacatccactaaggaggtgctggacgctaccctgattcaccagagcattaccggcctgtatgaaacaaggattgacctgtctcagctggggggcgactctggcggcagcaccaacctgagcgacatcatcgagaaagagacaggcaagcagctggtcatccaagagtccatcctgatgctgcctgaagaggtggaagaagtgatcggcaacaagcccgagtccgacatcctggtgcacaccgcctacgatgagagcaccgacgagaacgtgatgctgctgacctctgacgcccctgagtacaagccttgggctctcgtgatccaggacagcaacggcgagaacaagatcaagatgctgagcggcggcagccctaagaagaagcggaaggtcgaggaccccaagaaaaaacggaaggtgctcgaggatggggacgagggcagaggaagtcttctaacatgcggtgacgtcgaggagaatcctggcccagcaccgggatccatgagcgagctgatcaaggagaacatgcacatgaagctgtacatggagggcaccgtgaacaaccaccacttcaagtgcacatccgagggcgaaggcaagccctacgagggcacccagaccatgaagatcaaggtggtcgagggcggccctctccccttcgccttcgacatcctggctaccagcttcatgtacggcagcaaagccttcatcaaccacacccagggcatccccgacttctttaagcagtccttccctgagggcttcacatgggagagaatcaccacatacgaagacgggggcgtgctgaccgctacccaggacaccagcttccagaacggctgcatcatctacaacgtcaagatcaacggggtgaacttcccatccaacggccctgtgatgcagaagaaaacacgcggctgggaggccaacaccgagatgctgtaccccgctgacggcggcctgagaggccacagccagatggccctgaagctcgtgggcgggggctacctgcactgctccttcaagaccacatacagatccaagaaacccgctaagaacctcaagatgcccggcttccacttcgtggaccacagactggaaagaatcaaggaggccgacaaagagacctacgtcgagcagcacgagatggctgtggccaagtactgcgacctccctagcaaactggggcacagatgataatctagagggcccgtttaaacccgctgatcagcctcgactgtgccttctagttgccagccatctgttgtttgcccctcccccgtgccttccttgaccctggaaggtgccactcccactgtcctttcctaataaaatgaggaaattgcatcgcattgtctgagtaggtgtcattctattctggggggtggggtggggcaggacagcaagggggaggattgggaagacaatagcaggcatgctggggatgcggtgggctctatggcttctactgggcggttttatggacagcaagcgaaccggaattgccagctggggcgccctctggtaaggttgggaagccctgcaaagtaaactggatggctttcttgccgccaaggatctgatggcgcaggggatcaagctctgatcaagagacaggatgaggatcgtttcgcatgattgaacaagatggattgcacgcaggttctccggccgcttgggtggagaggctattcggctatgactgggcacaacagacaatcggctgctctgatgccgccgtgttccggctgtcagcgcaggggcgcccggttctttttgtcaagaccgacctgtccggtgccctgaatgaactgcaagacgaggcagcgcggctatcgtggctggccacgacgggcgttccttgcgcagctgtgctcgacgttgtcactgaagcgggaagggactggctgctattgggcgaagtgccggggcaggatctcctgtcatctcaccttgctcctgccgagaaagtatccatcatggctgatgcaatgcggcggctgcatacgcttgatccggctacctgcccattcgaccaccaagcgaaacatcgcatcgagcgagcacgtactcggatggaagccggtcttgtcgatcaggatgatctggacgaagagcatcaggggctcgcgccagccgaactgttcgccaggctcaaggcgagcatgcccgacggcgaggatctcgtcgtgacccatggcgatgcctgcttgccgaatatcatggtggaaaatggccgcttttctggattcatcgactgtggccggctgggtgtggcggaccgctatcaggacatagcgttggctacccgtgatattgctgaagagcttggcggcgaatgggctgaccgcttcctcgtgctttacggtatcgccgctcccgattcgcagcgcatcgccttctatcgccttcttgacgagttcttctgaattattaacgcttacaatttcctgatgcggtattttctccttacgcatctgtgcggtatttcacaccgcatacaggtggcacttttcggggaaatgtgcgcggaacccctatttgtttatttttctaaatacattcaaatatgtatccgctcatgagacaataaccctgataaatgcttcaataatagcacgtgctaaaacttcatttttaatttaaaaggatctaggtgaagatcctttttgataatctcatgaccaaaatcccttaacgtgagttttcgttccactgagcgtcagaccccgtagaaaagatcaaaggatcttcttgagatcctttttttctgcgcgtaatctgctgcttgcaaacaaaaaaaccaccgctaccagcggtggtttgtttgccggatcaagagctaccaactctttttccgaaggtaactggcttcagcagagcgcagataccaaatactgtccttctagtgtagccgtagttaggccaccacttcaagaactctgtagcaccgcctacatacctcgctctgctaatcctgttaccagtggctgctgccagtggcgataagtcgtgtcttaccgggttggactcaagacgatagttaccggataaggcgcagcggtcgggctgaacggggggttcgtgcacacagcccagcttggagcgaacgacctacaccgaactgagatacctacagcgtgagctatgagaaagcgccacgcttcccgaagggagaaaggcggacaggtatccggtaagcggcagggtcggaacaggagagcgcacgagggagcttccagggggaaacgcctggtatctttatagtcctgtcgggtttcgccacctctgacttgagcgtcgatttttgtgatgctcgtcaggggggcggagcctatggaaaaacgccagcaacgcggcctttttacggttcctgggcttttgctggccttttgctcacatgttcttgactcttcgcgatgtacgggccagatata

Sequence of NLS-**APOBEC3A**-XTEN linker-Cas9(D10A)-UGI-T2A-TurboRFP BE3

**gaagcctctcctgcctctggacccagacacctgatggaccctcacatcttcaccagcaacttcaacaacggcatcggccggcacaagacctacctgtgctacgaggtggaacggctggacaatggcaccagcgtgaagatggaccagcaccggggctttctgcacaaccaggccaagaatctgctgtgcggcttctacggcagacacgccgagctgagatttctggacctggtgcctagcctgcagctggaccctgctcagatctacagagtgacctggttcatcagctggtccccatgcttctcttggggctgtgctggcgaagtgcgcgccttcctgcaagagaatactcacgtgcggctgcggatcttcgccgccagaatctacgactacgaccctctgtacaaagaggccctgcagatgctgagagatgccggtgctcaggtgtccatcatgacctacgacgagttcaagcactgctgggacaccttcgtggatcaccagggctgccctttccagccttgggatggactggatgagcactctcaggccctgagcggtagactgagagccatcctgcagaaccagggcaac**

Sequence of NLS-**APOBEC3B**-XTEN linker-Cas9(D10A)-UGI-T2A-TurboRFP BE3

**gagatcctgagatacctgatggaccccgacaccttcaccttcaatttcaacaacgaccctctggtgctgcggcggagacagacctacctgtgttacgaggtggaacggctggacaacggcacctgggttctgatggatcagcacatgggctttctgtgcaacgaggccaagaacctgctgtgcggcttctatggcagacacgccgagctgcggtttctggatctggtgccttctctgcagctggaccccgctcagatctacagagtgacctggttcatcagctggtccccatgcttctcttggggctgtgctggcgaagtgcgggccttcctgcaagagaatactcacgtgcggctgcggatcttcgccgccagaatctacgactacgatcccctgtacaaagaggccctgcagatgctgagagatgccggtgctcaggtgtccatcatgacctacgacgagttcgagtactgctgggacaccttcgtgtacagacagggctgccctttccagccttgggacggactggaagaacactctcaggctctgagcggcagactgagagccattctt**

Sequence of NLS-**rA1 (no codon optimisation)**-XTEN linker-Cas9(D10A)-UGI-T2A-TurboRFP BE3

**agttccgagacaggccctgtagctgttgatcccactctgaggagaagaattgagccccacgagtttgaagtcttctttgacccccgggaacttcggaaagagacctgtctgctgtatgagatcaactggggaggaaggcacagcatctggcgacacacgagccaaaacaccaacaaacacgttgaagtcaatttcatagaaaaatttactacagaaagatacttttgtccaaacaccagatgctccattacctggttcctgtcctggagtccctgtggggagtgctccagggccattacagaatttttgagccgatacccccatgtaactctgtttatttatatagcacggctttatcaccacgcagatcctcgaaatcggcaaggactcagggaccttattagcagcggtgttactatccagatcatgacggagcaagagtctggctactgctggaggaattttgtcaactactccccttcgaatgaagctcattggccaaggtacccccatctgtgggtgaggctgtacgtactggaactctactgcatcattttaggacttccaccctgtttaaatattttaagaagaaaacaacctcaactcacgtttttcacgattgctcttcaaagctgccattaccaaaggctaccaccccacatcctgtgggccacagggttgaaa**
